# Comparison between the analytical sensitivity and clinical performance of two cobas SARS-CoV-2 tests based on high-throughput and point-of-care systems

**DOI:** 10.37796/2211-8039.1334

**Published:** 2022-06-01

**Authors:** Tsai-Hsiu Lin, Ngoc-Niem Bui, Yu-Chang Chang, Li-Yun Hsu, Yang-Di Su, Chieh-Min Chang, Wei-An Hong, Uyen Nguyen Phuong Le, Su-Hua Huang, Cheng-Wen Lin

**Affiliations:** a Department of Laboratory Medicine, China Medical University Hospital, Taichung 40402, Taiwan; b Department of Medical Laboratory Science and Biotechnology, China Medical University, Taichung 40402, Taiwan; c Faculty of Medicine, Can Tho University of Medicine and Pharmacy, Can Tho 94117, Viet Nam; d Department of Medical Laboratory Science and Biotechnology, Asia University, Taichung 41354, Taiwan; e Graduate Institute of Biological Science and Technology, China Medical University, Taichung 40402, Taiwan

**Keywords:** SARS-CoV-2 test, Cobas 6800, Cobas liat, Analytical sensitivity, Clinical performance

## Abstract

**Objectives:**

This study examined analytical sensitivity, specificity, and the clinical performance in detecting SARS-CoV-2 of the Cobas SARS-CoV-2 Test based on the high-throughput Cobas 6800 system and the Cobas SARS-CoV-2 & Flu A/B Test based on the point-of-care cobas Liat system.

**Methods:**

The commercial reagents containing SARS-CoV-2 RNA subgenomes were diluted for assessing the sensitivity of the RT-qPCR assay. 385 nasopharyngeal swab specimens taken from contacts of COVID-19 cases were tested for the SARS-CoV-2 detection with both Cobas SARS-CoV-2 Tests.

**Results:**

In analytical sensitivity assays, the Cobas SARS-CoV-2 & Flu A/B Test on the Liat system had a lower limit of detection (12.5–25 copies/mL) than the cobas SARS-CoV-2 Test on the cobas 6800 system (25–50 copies/mL). In clinical performance assays, the cobas SARS-CoV-2 Test demonstrated 89.36% (42 out of 47) PPA (positive percent agreement) and 98.82% (334 out of 338) NPA (negative percent agreement) compared to the results of the Cobas SARS-CoV-2 & Flu A/B test. Among five discordant specimens, four had the positive result of the cobas SARS-CoV-2 test, but the negative result of the cobas SARS-CoV-2 & Flu A/B Test. Moreover, these discordant specimens had the Ct values of greater than 33 for the cobas SARS-CoV-2 Test, implying a very small number of virions in the samples. Remarkably, four specimens with a presumptive positive result of the cobas SARS-CoV-2 test had been confirmed by the Cobas SARS-CoV-2 & Flu A/B Test. Next, the scatter plots of the Ct values showed a highly positive correlation between cobas SARS-CoV-2 & Flu A/B Test and the cobas SARS-CoV-2 Test (R-squared value = 0.954–0.962).

**Conclusions:**

Both SARS-CoV2 tests of the cobas 6800 and Liat systems produce reliable high throughput and point-of-care assays respectively for the early virus detection and the personal care decision-making during COVID-19 pandemic.

## 1. Introduction

Severe acute respiratory syndrome coronavirus type 2 (SARS-CoV-2), causing coronavirus disease 2019 (COVID-19), is very contagious and rapidly spreads across the globe [1], which poses significant challenges to public health and the economy [2]. The pandemic is on-going and there is barely any effective treatment found, social and physical distancing and self-quarantine have become crucial methods in the struggle to block the SARS-CoV-2 transmission cycle [3]. However, the first step in managing COVID-19 is rapid and accurate detection to prevent infectious spread. Rapid SARS-CoV-2 diagnostic tests are critical for prompt diagnosis and treatment and are necessary to detect the individuals who carry the virus without symptoms [4]. Real-time reverse-transcription polymerase chain reaction (RT-PCR) is a rapid, available, and reliable tool for developing diagnostic tests to detect and quantitate SARS-CoV-2 nucleic acids in respiratory tract specimens from the people who have asymptomatic, pre-symptomatic, or symptomatic COVID-19 [4,5]. World Health Organization (WHO) releases some RT-PCR protocol assays for detecting SARS-CoV-2 genes, including RNA-dependent RNA polymerase (RdRp), envelope (E), spike (S), open reading frame (ORF) 1a/b, and nucleocapsid (N). The CDC in the US also obtains the emergency use authorization (EUA) from FDA (Food and Drug Administration) for the first RT-PCR test. During the epidemic, a growing number of commercial kits for detecting SARS-CoV-2 and its variations were created, which were then employed in clinical laboratories and other organizations [6–8].

Roche’s Cobas 6800 system, an automated two-target RT-PCR testing technology for SARS-CoV-2 genes ORF1a/b and E, was granted an Emergency Use Authorization (EUA) by the US Food and Drug Administration (FDA) on March 12th, 2020, to meet the demands of diagnostic kits when there was no sign of the pandemic situation to improve [3,9]. The cobas 6800 assay is the automated system for sample preparation (nucleic acid extraction and purification) following Real-time PCR amplification and detection. The cobas SARS-CoV-2 on Cobas 6800 System is a dual-target design on ORF1a/b and E genes under two distinct channels detection. It was reported as positive, reactive, and negative according to the results of ORF1 a/b and E targets detected [10]. The cobas SARS-CoV-2 & Influenza A/B nucleic acid Test performed on the Cobas Liat Analyzer, an automated multiplex real-time RT-PCR platform, has also been authorized by FDA to detect SARS-CoV-2, influenza A, and influenza B virus RNA in nasopharyngeal and nasal specimens in authorized laboratories [10–12]. The cobas SARS-CoV-2 and influenza A/B nucleic acid Test on the Cobas Liat Analyzer is designed based on the multiplex RT-PCR kit that automates NAAT processes for influenza A/B and RSV pathogens. Under the COVID-19 pandemic, the Cobas SARS-CoV-2 and influenza A/B kit identifies SARS-CoV-2 RdRp and N genes under single-channel detection. The materials provided for multiplex PCR and the internal process control (IPC) have been combined into a single tube kit, that may be used at the point of care (POC) or in a clinical laboratory setting [11]. The Liat SARS-CoV-2 and influenza A/B assay automates processes such as target enrichment, inhibitor removal, nucleic acid extraction, amplification, real-time detection, and result in 20 min per sample [11,12].

The new wave of COVID-19 transmission flooding into Taiwan in mid-May 2021 raised the concerns in upgrading the platform of high-throughput cobas 6800 and POC Cobas Liat systems with SARS-CoV-2 detection tests. This study evaluated analytical sensitivity, specificity, and the clinical performance of two singleplex RT-PCR assays (Cobas SARS-CoV-2 Test) on Cobas 6800 system and one multiplex RT-PCR assay (Cobas SARS-CoV-2 & Flu A/B Test) on a Liat system for the diagnosis of SARS-CoV-2 infection.

## 2. Materials and methods

### 2.1. Control material and analytical sensitivity

AccuPlex™ SARS-CoV-2 Reference Material Kit (material number: 0505-0126) was used as the SARS-CoV-2 reference standard material. The kit contains 5000 copies/ml RNA materials with a viral protein coat, comprising the nucleotides of SARS-CoV-2 sequence 417-1899, 3094-3360 for ORF1a, 13291-13560, 14700-15950, 18577-19051 for RdRp, 25801-28200 for E, and 27952-29873 for N. To assess the sensitivity of the RT-qPCR assay, the positive RNA materials with a viral protein coat in above kit were serially diluted, extracted, and then performed by the protocols of both SARS-CoV-2 detection tests. The LOD is the lowest concentration of SARS-CoV-2 RNA that can be detected in >95% of samples tested with acceptable precision by our systems. To compare the LOD among two systems, the two-fold dilutions were prepared and performed in triplicates using Cobas SARS-CoV-2 Test on Cobas 6800 System and Cobas SARS-CoV-2 & Flu A/B Test on Cobas Liat System according to the manuals of manufacturers’ instruction. The mean value and standard deviation (SD) were calculated regarding the Ct values of targets either the ORF1 a/b and E genes for singleplex RT-PCR assays (Cobas SARS-CoV-2 Test) or RdRp and N genes for multiplex RT-PCR (Cobas SARS-CoV-2 & Flu A/B Test). The result of both SARS-CoV-2 detection tests was reported as positive and negative according to manufacturers’ instructions.

### 2.2. Analytical specificity

The analytical specificity of Cobas SARS-CoV-2 Test on Cobas 6800 System and Cobas SARS-CoV-2 & Flu A/B Test on Cobas Liat System was determined by examining 20 common respiratory viruses, including Coxsackie A9, Coxsackie A16, Coxsackie A24, Coxsackie B1 to B5, Influenza A/B, Parainfluenza 1/2/3, EV71, Echo 4/6/9/11/30, ECHO4.

### 2.3. Clinical specimen and real-time RT PCR assays

All nasopharyngeal swab specimens were collected from employees at an electronic company in Zhunan Industry Park in Taiwan from May 31st to June 5th, 2021. The swabs were well placed in a disposable virus sampling tube containing a 3 mL transport medium and delivered to the clinical laboratory at China Medical University Hospital (CMUH), Taiwan. All samples were performed using the assays with Cobas SARS-CoV-2 Test on Cobas 6800 System and Cobas SARS-CoV-2 & Flu A/B Test on Cobas Liat System according to the manuals of manufacturers’ instruction. The cycle threshold (Ct) values were reported based on valid test results of both tests.

### 2.4. Statistical analysis

From the Ct value analysis of matched specimens detected by Cobas SARS-CoV-2 kits of Cobas 6800 and Liat testing systems, the overall percent agreement (OPA), PPA (positive percent agreement), and NPA (negative percent agreement) were tested by kappa statistics and SPSS simple linear regression analysis of SPSS respectively.

## 3. Results

### 3.1. Higher analytical sensitivity of SARS-CoV-2 RdRp and N-based duplex RT-PCR assay on Cobas Liat system

In order to evaluate the analytical sensitivity and specificity of the Cobas SARS-CoV-2 Test and the Cobas SARS-CoV-2 & Flu A/B Test, serial dilutions of the commercial control containing the SARS-CoV-2 RNA genome were tested with high-throughput Cobas 6800 and POC Cobas Liat systems, respectively (Tables 1 and 2). The concentration level observing hit rates is either greater than or equal to 95% at 50 copies/ml for both single targets of SARS-CoV-2 ORF1a/b gene or SARS-CoV-2 E gene by the Cobas SARS-CoV-2 Test on Cobas 6800 system. In terms of higher than 95% hit rates, the analytical sensitivity of the Liat system with the combined detection of RdRP and N gene in the Cobas SARS-CoV-2 & Flu A/B Test was roughly 25 copies of RNA genome equivalent per reaction. The analytical detection limit of the Cobas SARS-CoV-2 Test on the Cobas 6800 system was between 25 and 50 copies/mL for detecting a single target gene ORF1a/b or E. Meanwhile, the lower detection limit for the Cobas SARS-CoV-2 & FluA/B Test on the Liat system was between 12.5 and 25 copies/ml (Table 1). The result indicated that multiplex RT-PCR assay of Cobas SARS-CoV-2 & Flu A/B Test on Liat system had a greater analytical sensitivity of SARS-CoV-2 detection than two singleplex RT-PCR reactions of the Cobas SARS-CoV-2 Test channels on Cobas 6800 system.

In order to evaluate the analytical specificity of Cobas SARS-CoV-2 Test and Cobas SARS-CoV-2 & Flu A/B Test, the specimens of 20 common viruses were tested with both tests (Table 2). These are all prevalent viruses with no cross-reactivity, which showed the high specificity of SARS-CoV-2 detection by the Cobas SARS-CoV-2 Test on Cobas 6800 and cobas SARS-CoV-2 & Flu A/B Test on Liat systems.

### 3.2. Clinical performance comparison between the two systems

**Fig. 1:**
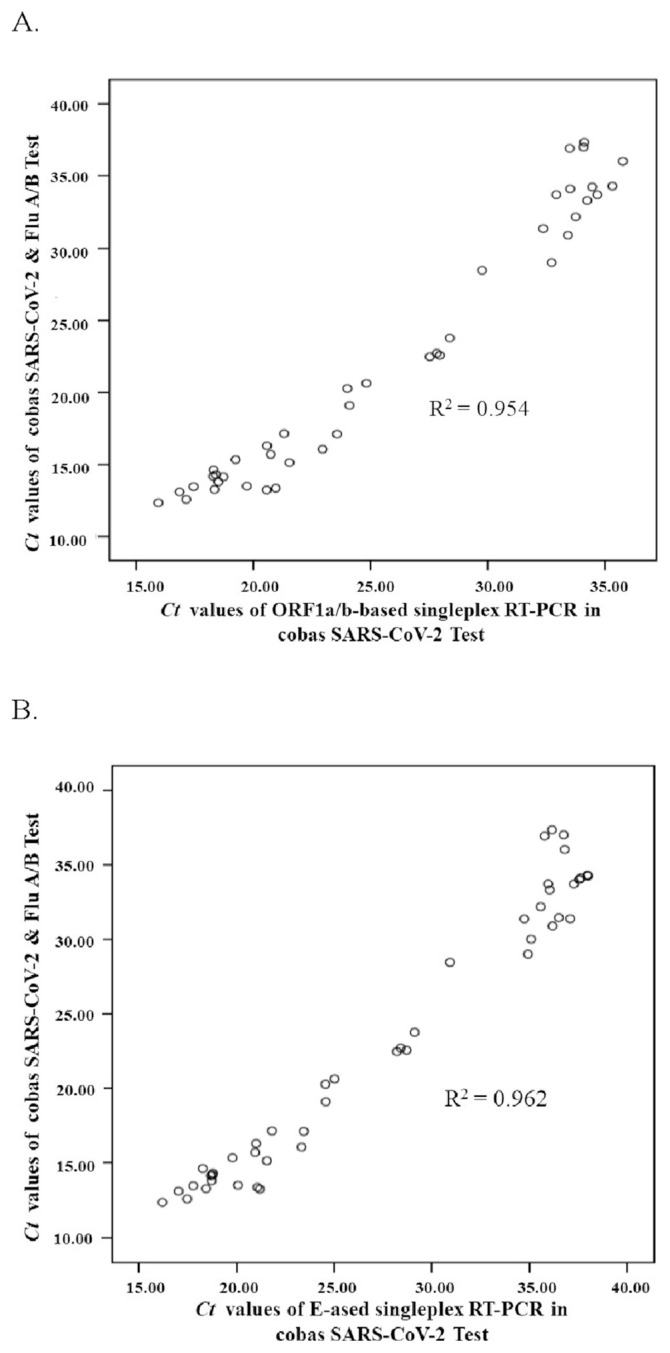
Correlation between cycle threshold (Ct) values obtained from real-time RT-PCR assays in both Cobas tests. The scatter plot posed the Ct values generated from ORF1a/b-targeted singleplex RT-PCR reactions of the Cobas SARS-CoV-2 Test compared to the cobas SARS-CoV-2 & Flu A/B Test (A). The scatter plot represented the Ct values generated from E-targeted singleplex RT-PCR reactions of the Cobas SARS-CoV-2 Test compared to the cobas SARS-CoV-2 & Flu A/B Test (B).

To compare the clinical performance of the cobas SARS-CoV-2 Test on high throughput platform and POC assays, the Cobas SARS-CoV-2 Test on Cobas 6800 and cobas SARS-CoV-2 & Flu A/B Test on Liat systems were further performed for the diagnosis of the SARS-CoV-2 outbreak at an electronic company in Zhunan Industry Park in Taiwan from May 31st to June 5th, 2021. A total of 385 nasopharyngeal swab specimens from contacts of COVID-19 cases were performed for the SARS-CoV-2 detection using Cobas SARS-CoV-2 Test on Cobas 6800 and cobas SARS-CoV-2 & Flu A/B Test on Liat systems. Both tests yielded nearly identical results for detecting almost all specimens. On May 31st, four samples had a high Ct Value in the cobas SARS-CoV-2 Test on the Cobas 6800, but were undetectable in the cobas SARS-CoV-2 & Flu A/B Test on the Liat system; 1 sample (June 1st) presented a high Ct value of cobas SARS-CoV-2 & Flu A/B Test on Liat system but was undetectable by the Cobas SARS-CoV-2 Test on Cobas 6800 system. Comparing to the Cobas SARS-CoV-2 Test on Cobas 6800 systems as the reference method fot the SARS-CoV-2 detection, the clinical performance evaluation of the cobas SARS-CoV-2 & Flu A/B Test on Liat system revealed 92% (46 out of 50) PPA (positive percent agreement) and 99.7% (334 out of 335) NPA (negative percent agreement) (Table 3). There were 5 specimens with conflicting results out of 385 tested (Table 4). The Cobas SARS-CoV-2 Test found four specimens to be positive, while the Cobas SARS-CoV-2 & Flu A/B Test found them to be negative. One specimen was negative by for Cobas SARS-CoV-2 Test, but positive by for cobas SARS-CoV-2 & Flu A/B Test. Both tests revealed a Ct value greater than 33 in these 5 discordant specimens, indicating a reduced viral content in these specimens. The scatter plots with Pearson correlation and linear regression analysis were created using SPSS Statistics to evaluate the correlation between Ct values of singleplex RT-PCR in the Cobas SARS-CoV-2 Test and multiplex RT-PCR in the Cobas SARS-CoV-2 & Flu A/B Test (Fig. 1). The scatter diagram illustrated that the Ct values for the cobas SARS-CoV-2 & Flu A/B Test had a higher degree of the positive correlation with the Ct values of E-targeted singleplex RT-PCR assay of the cobas SARS-CoV-2 Test (r-square = 0.962) than ORF1a/b-targeted singleplex RT-PCR assay of the cobas SARS-CoV-2 Test (r-square = 0.954). Furthermore, the scatter plot revealed that the mean Ct shifts were 4.36 ± 1.97 for E-targeted singleplex RT-PCR assay, and 3.22 ± 2.78 for ORF1a/b-targeted singleplex RT-PCR assay of the Cobas SARS-CoV-2 Test compared to the cobas SARS-CoV-2 & Flu A/B Test.

## 4. Discussion

While existing COVID-19 vaccines are being used, the rapid spread of SARS-CoV-2 may have aided the generation of variations that can at least partially evade the human immune system and reappear on a regular basis [13,14]. This concern has provided an impetus to increase the tests of SARS-CoV-2 detection in illness patients and predominantly asymptomatic infection to promote viral spread. Following the FDA EUAs, the manufacturers demonstrated the measurement of commercial tests analytical and clinical test performance [9]. The initial step, as stated in several standards for validation and verification of nucleic assays (CLSI, ISO 15189), is to concentrate on target gene selection [15,16]. The ORF1a/b and E genes were chosen as targets for two singleplex RT-PCR assay of distinct channels in the cobas®SARS-CoV-2 commercial kit on Cobas 6800 system, in which the ORF1a/b primer binding sequence was unique toSARS-CoV-2, and a conserved region in the E gene was chosen for pan-Sarbecovirus detection [17]. The target genes for multiplex RT-PCR under single-channel detection in the SARS-CoV-2&Flu A/B Test on the Cobas Liat Analyzer were SARS-CoV-2 RdRpandNgenes [11]. Of the 385 specimens tested in this study, the positive rates were 12.99% for the Cobas SARS-CoV-2 Test on Cobas 6800 system, and 12.21% for the cobas SARS-CoV-2 & Flu A/B Test on Liat system, respectively (Table 3). The clinical performance evaluation indicated that cobas SARS-CoV-2 & Flu A/B Test on Liat system had 92% (46 out of 50) PPA (positive percent agreement) and 99.7% (334 out of 335) NPA (negative percent agreement) compared to the Cobas SARS-CoV-2 Test on Cobas 6800 systems (Table 3). Pearson correlation and linear regression analysis of Ct values for individual samples revealed a very strong positive correlation between both the two SARS-CoV-2 tests, with r-squared values better than 0.95 in the scatter plots. As a result, the study found that both SARS-CoV-2 tests on the Cobas 6800 and Liat systems were clinically valid and trustworthy.

Four of the five conflicting specimens reported Ct values ranging from 33.75 to 37.71 for the Cobas SARS-CoV-2 Test on the Cobas 6800 system, while one had a Ct value of 33.14 for the Cobas SARS-CoV-2 & Flu A/B Test on the Cobas Liat system. The result revealed a lower viral concentration in these discordant specimens, which was around the limit of detection of the test. Similarly, four presumptive positive specimens were positive for E gene target, but negative for ORF1a/b target detected by the Cobas SARS-CoV-2 test of the Cobas 6800 system, while being positive as detected by the Cobas SARS-CoV-2 & Flu A/B Test of Cobas Liat system (Table 4). For E target singleplex RT-PCR assay, these 4 presumptive positive specimens had significantly delayed Ct values ranging from 35.09 to 37.55, indicating a minimum amount of virions that might be under the limit of detection for ORF1a/b target singleplex RT-PCR assay. Therefore, E target singleplex RT-PCR assay could have a higher clinical performance than ORF1a/b target gene singleplex RT-PCR assay of the Cobas SARS-CoV-2 Test on the Cobas 6800 system.

Comparing to the Cobas SARS-CoV-2 Test on the Cobas 6800 system, the Cobas SARS-CoV-2 & Flu a/B Test on the Cobas Liat system had higher analytical sensitivity and a lower limit of detection (Table 1). Meanwhile, the cobas SARS-CoV-2 & Flu A/B Test exhibited significantly lower mean Ct values of individual positive samples than the cobas SARS-CoV-2 Test, which had a mean Ct shift of 4.36 ± 1.97 following E-targeted singleplex RT-PCR assay (Table 1, Fig. 1). Nevertheless, the Cobas SARS-CoV-2 & Flu A/B Test of the Cobas Liat system had a lower clinical sensitivity compared with the Cobas SARS-CoV-2 Test of the Cobas 6800 system (Table 3), this could be related to the impact of the nucleic acid extraction process on the removal of PCR inhibitors from clinical samples, resulting in a reduction of clinical sensitivity. The Cobas SARS-CoV-2 Test and Cobas SARS-CoV-2 & Flu A/B Test are reliable assays for the qualitative detection of SARS-CoV-2 in the clinical laboratory during the pandemic outbreak, when faced with many specimens from symptomatic and asymptomatic patients. Moreover, cobas SARS-CoV-2 & Flu A/B Test on the Liat system may provide a solution for rapidly confirming cases with the reactive result of the cobas SARS-CoV-2 Test on Cobas 6800 system.

## 5. Conclusions

While the world wait for the cover of vaccination and the novel effective antiviral medicals, SARS-CoV-2 is continuing to spread rapidly, and there is high probabilities of new varieties occurring in different locations over the globe. The enhanced surveillance of new variants could affect the diagnostic testing regimens. Our findings demonstrate that both tests, including the Cobas SARS-CoV-2 & Flu A/B Test on Liat system and the Cobas SARS-CoV-2 Test on the Cobas 6800 assays, have good performance characteristics and are reliable assays for detecting SARS-CoV-2 in a qualitative and quantitative manner. This study also reveals that both high throughput and POC SARS-CoV-2 tests have strong analytical sensitivity and clinical performance, assisting early infection management and personal care decisions during the COVID-19 outbreak considerably.

## Figures and Tables

**Table 1:** Sensitivity comparison of the cobas SARS-CoV-2 test on cobas 6800 system and the cobas SARS-CoV-2 & Flu A/B test on Liat system.

Concentration (copies/mL)	ORF1-targeted singleplex RT-PCR assay in Cobas SARS-CoV-2 Test		E– targeted singleplex RT-PCR assay in Cobas SARS-CoV-2 Test		RdRp and N-based multiplex RT-PCR in Cobas SARS-CoV-2 & Flu A/B Test	
		
	Hit rate [%]	Ct (mean ± SD)	Hit rate [%]	Ct (mean ± SD)	Hit rate [%]	Ct (mean ± SD)
100	100	35.03 ± 0.55	100	37.12 ± 0.35		
50	100	36.04 ± 0.58	100	38.01 ± 0.77	100	33.87 ± 1.25
25	75	36.54 ± 0.38	88	39.72 ± 2.49	100	34.67 ± 1.09
12.5	63	36.56 ± 0.35	50	39.29 ± 0.40	88.3	35.83 ± 0.22
6.25					60	35.28 ± 1.05

**Table 2:** Specificity evaluation of the cobas SARS-CoV-2 test on cobas 6800 system and the cobas SARS-CoV-2 & Flu A/B test on Liat system.

Virus type	cobas SARS-CoV-2 Test			cobas SARS-CoV-2 & Flu A/B Test

	ORF1 gene	E gene	Internal control	
coxsackie A24	Negative	Negative	Valid	Negative
Coxsackie A16	Negative	Negative	Valid	Negative
Influenza B	Negative	Negative	Valid	Positive
Influenza A	Negative	Negative	Valid	Positive
Parainfluenza 2	Negative	Negative	Valid	Negative
Parainfluenza 1	Negative	Negative	Valid	Negative
Parainfluenza 3	Negative	Negative	Valid	Negative
EV71	Negative	Negative	Valid	Negative
Coxsackie A9	Negative	Negative	Valid	Negative
ECHO4	Negative	Negative	Valid	Negative
Echo 11	Negative	Negative	Valid	Negative
Echo 30	Negative	Negative	Valid	Negative
Echo 6	Negative	Negative	Valid	Negative
Echo 4	Negative	Negative	Valid	Negative
Echo 9	Negative	Negative	Valid	Negative
Coxsackie B2	Negative	Negative	Valid	Negative
coxsackie B1	Negative	Negative	Valid	Negative
Coxsackie B5	Negative	Negative	Valid	Negative
Coxsackie B4	Negative	Negative	Valid	Negative
Coxsackie B3	Negative	Negative	Valid	Negative

**Table 3:** Detection of 385 nasopharyngeal specimens using the cobas SARS-CoV-2 test on cobas 6800 system and the cobas SARS-CoV-2 & Flu A/B test on Liat system.

	cobas SARS-CoV-2 Test		Total	Kappa value	*P-*value

	Detected	Not-detected			
**cobas SARS-CoV-2**				0.941	0.000
& **Flu A/B Test**					
Detected	46	1	47		
Not-detected	4	334	338		
Total	50^a^	335			

a All target results were valid in the reaction with negative ORF1a/b gene and positive E gene following the Cobas SARS-CoV-2 test.

**Table 4:** Comparison of Ct values among discordant specimens detected by the cobas SARS-CoV-2 test and cobas SARS-CoV-2 & Flu A/B test.

Sample Bar-Code	Cobas SARS-CoV-2 Test			SARS-CoV-2 & Flu A/B Test	
	
	ORF1a/b gene (Ct value)	E gene (Ct value)	Result	RdRp/N gene (Ct value)	Result
06-109-266160	34.94	37.71	Positive	TND	Negative
06-109-266169	34.09	35.76	Positive	TND	Negative
06-109-266179	33.75	36.58	Positive	TND	Negative
06-109-266183	34.27	35.9	Positive	TND	Negative
06-109-265920	TND^a^	35.09	Reactive^b^	30	Positive
06-109-331129	TND	36.51	Reactive	31.44	Positive
06-109-266177	TND	37.09	Reactive	31.37	Positive
06-109-330219	TND	TND	Negative	33.14	Positive
06-109-266577	TND	37.55	Reactive	34	Positive

a TND, target not detected.

b All Target Results were valid.

Result for SARS-CoV-2 RNA is Presumptive Positive.

## References

[1] Cobey S Larremore DB Grad YH Lipsitch M Concerns about SARS-CoV-2 evolution should not hold back efforts to expand vaccination Nat Rev Immunol 2021 21 5 330 5 33795856 10.1038/s41577-021-00544-9 PMC8014893

[2] Tegally H Wilkinson E Giovanetti M Iranzadeh A Fonseca V Giandhari J Detection of a SARS-CoV-2 variant of concern in South Africa Nature 2021 592 7854 438 43 33690265 10.1038/s41586-021-03402-9

[3] You HL Lin MC Lee CH Comparison of the Roche cobas 6800 SARS-CoV-2 test and the Taiwan CDC protocol for the molecular diagnosis of COVID-19 Biomed J 2021 44 1 101 4 33736952 10.1016/j.bj.2020.12.007 PMC7771907

[4] Rothe C Schunk M Sothmann P Bretzel G Froeschl G Wallrauch C Transmission of 2019-nCoV infection from an asymptomatic contact in Germany N Engl J Med 2020 March 5 382 10 970 1 32003551 10.1056/NEJMc2001468 PMC7120970

[5] Kevadiya BD Machhi J Herskovitz J Oleynikov MD Blomberg WR Bajwa N Diagnostics for SARS-CoV-2 infections Nat Mater 2021 20 5 593 605 33589798 10.1038/s41563-020-00906-z PMC8264308

[6] Bloom JS Sathe L Munugala C Jones EM Gasperini M Lubock NB Massively scaled-up testing for SARS-CoV-2 RNA via next-generation sequencing of pooled and barcoded nasal and saliva samples Nat Biomed Eng 2021 5 7 657 65 34211145 10.1038/s41551-021-00754-5 PMC10810734

[7] Bustin S Coward A Sadler G Teare L Nolan T CoV2-ID, a MIQE-compliant sub-20-min 5-plex RT-PCR assay targeting SARS-CoV-2 for the diagnosis of COVID-19 Sci Rep 2020 10 1 22214 33335187 10.1038/s41598-020-79233-x PMC7747624

[8] Davi MJP Jeronimo SMB Lima JPMS Lanza DCF Design and in silico validation of polymerase chain reaction primers to detect severe acute respiratory syndrome coronavirus 2 (SARS-CoV-2) Sci Rep 2021 11 1 12565 34131209 10.1038/s41598-021-91817-9 PMC8206341

[9] Pujadas E Ibeh N Hernandez MM Waluszko A Sidorenko T Flores V Comparison of SARS-CoV-2 detection from nasopharyngeal swab samples by the Roche Cobas 6800 SARS-CoV-2 test and a laboratory-developed real-time RT-PCR test J Med Virol 2020 92 9 1695 8 32383179 10.1002/jmv.25988 PMC7267546

[10] Poljak M Korva M Knap Gašper N Fujs Komloš K Sagadin M Uršič T Clinical evaluation of the cobas SARS-CoV-2 test and a diagnostic platform switch during 48 hours in the midst of the COVID-19 pandemic J Clin Microbiol 2020 58 6 e00599 20 32277022 10.1128/JCM.00599-20 PMC7269406

[11] Hansen G Marino J Wang ZX Beavis KG Rodrigo J Labog K Clinical performance of the point-of-care cobas Liat for detection of SARS-CoV-2 in 20 minutes: a multicenter study J Clin Microbiol 2021 59 2 e02811 20 33239382 10.1128/JCM.02811-20 PMC8111162

[12] Maignan M Viglino D Hablot M Termoz Masson N Lebeugle A Collomb Muret R Diagnostic accuracy of a rapid RT-PCR assay for point-of-care detection of influenza A/B virus at emergency department admission: a prospective evaluation during the 2017/2018 influenza season PLoS One 2019 14 5 e0216308 31063477 10.1371/journal.pone.0216308 PMC6504036

[13] Rana K Mohindra R Pinnaka L Vaccine breakthrough infections with SARS-CoV-2 variants N Engl JMed 2021 July 8 385 2 e7 10.1056/NEJMc2107808 34077641

[14] Krause PR Fleming TR Longini IM Peto R Briand S Heymann DL SARS-CoV-2 variants and vaccines N Engl J Med 2021 385 2 179 86 34161052 10.1056/NEJMsr2105280 PMC8262623

[15] Raymaekers M Smets R Maes B Cartuyvels R Checklist for optimization and validation of real-time PCR assays J Clin Lab Anal 2009 23 3 145 51 19455629 10.1002/jcla.20307 PMC6649018

[16] Smith M Validating real-time polymerase chain reaction (PCR) assays Encycl Virol 2021 2021 35 44

[17] Dust K Hedley A Nichol K Stein D Adam H Karlowsky JA Comparison of commercial assays and laboratory developed tests for detecting SARS-CoV-2 J Virol Methods 2020 285 113970 32920028 10.1016/j.jviromet.2020.113970 PMC7482591

